# Electrically Enhanced Self-Thermophoresis of Laser-Heated Janus Particles under a Rotating Electric Field

**DOI:** 10.1038/s41598-018-24343-w

**Published:** 2018-04-13

**Authors:** Yu-Liang Chen, Cheng-Xiang Yang, Hong-Ren Jiang

**Affiliations:** Institute of Applied Mechanics, National Taiwan University. No.1, Sec. 4, Roosevelt Rd., Da’an Dist., Taipei City 106, Taiwan R.O.C.

## Abstract

The motion of a laser-heated Janus particle is experimentally measured under a rotating electric field. Directionally circular motions of the Janus particle following or countering the direction of the rotating electric field are observed in the low-frequency region (from 1 to 6 kHz) depending on the direction of electrorotation. In the higher frequency region (>10 kHz), only pure electrorotation and electrothermal flow are observed. By measuring the dependence of the frequency, voltage, and laser heating power, we propose that the tangential component of circular motion is caused by electric field enhanced self-thermophoresis, which is proportional to the laser heating power and the electric field. This result indicates that thermophoresis could be modified by the induced zeta potential of the Janus particle tuned by the applied electric fields. By this mechanism, the intrinsic thermophoresis can be enhanced several times at a relatively low applied voltage (~3 Volt). Electrically tunable thermophoresis of a particle may bring new insights to thermophoresis phenomenon and also open a new direction for tunable active materials.

## Introduction

Self-propulsion has recently received significant attention because of its potential practical applications in lab-on-chip devices^1^, drug delivery^2^, and its fundamental theoretical significance in non-equilibrium transport phenomena^3–5^. Self-propulsion provides an effective and promising strategy to design artificial active particles. Active particles driven by thermal gradients^3,6,7^, electric fields^5,8–10^ and chemical reactions^4,11^ have been demonstrated in various studies and this has become a promising field for building small active components for different applications, including microswimmers, nanomachines, and microrotors. This kind of motion often depends on the non-equilibrium response to its physical property. On the contrary, the coupling effect combining two physical fields is widely used in manipulating of micro-systems by locally changing the physical properties of one field and then driven by other fields such as electrothermal flow^12–14^. It would be useful if the properties of active particles could be dynamically tuned by one field and driven by the other. Several studies suggest that thermophoresis depends on the surface zeta potential of colloids^15–18^. According to previous studies, with the help of an electric field, additional zeta potential can be induced on polarizable objects such as Janus particles; this is called induced zeta potential^19,20^. Induced zeta potential has been proposed as the main mechanism of induced-charge effects for polarizable objects, such as induced-charge electrophoresis (ICEP) and induced-charge electro-osmosis (ICEO). These results suggest that it should be possible to enhance the thermophoresis of metallically coated Janus particles using an external electric field. Recently the electrorotation (EROT) of Janus particles (particles containing two distinct surfaces on two sides^21^), has been measured^10^, which shows that the direction of rotation depends on the frequency of the electric field. In EROT experiments, Janus particles exhibit pure rotational behavior without ICEP motion. This system may provide good conditions to investigate the enhancement of thermophoresis without the interference from ICEP. In this study, 2.34 μm Au-silica Janus particles are utilized as active particles to measure the self-thermophoresis under a rotating electric field. We find a new kind of active motion, which confirms that self-thermophoresis can be tuned by electric fields.

## Results

### Motion of Janus particles under a thermal-electric coupling field

**Figure 1:**
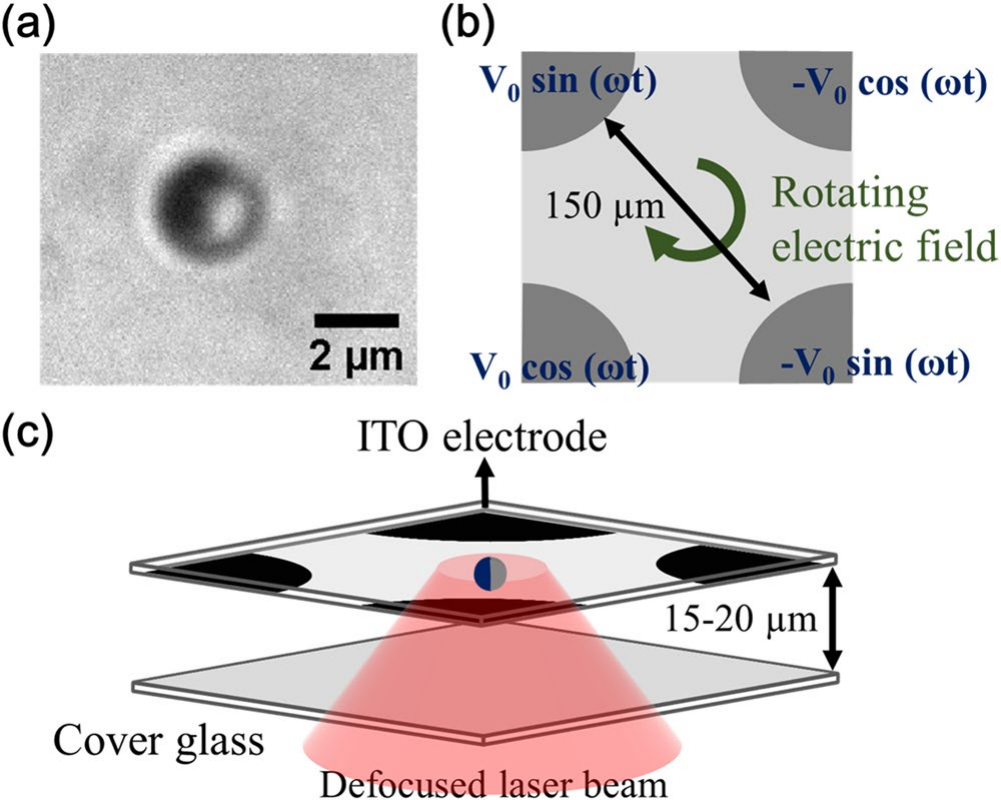
(**a**) Bright-field image of a Janus particle under a microscope. The dark side of the particle is the Au coating. (**b**) Schematics of the ITO four-phase electrode. The distance between the diagonal electrodes is 150 μm and the phase shift between the adjacent electrodes is set at 90 degrees. (**c**) Schematics of the experimental chamber (not drawn to scale). A thin chamber (15–20 μm) containing a solution and particles is sandwiched by a cover glass (bottom) and an ITO electrode (top). The defocused laser beam (diameter ~15–20 μm), red cone, is fed from the bottom.

**Figure 2:**
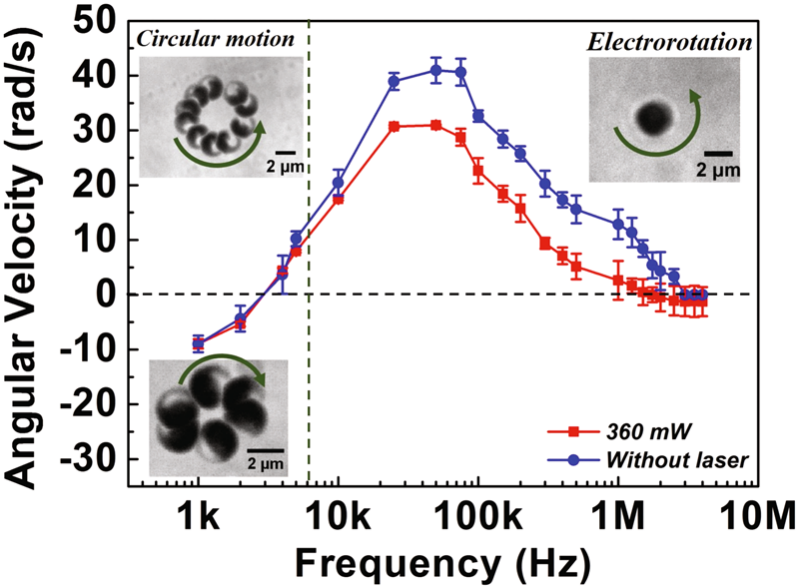
The relation between angular velocity and frequency of electric fields at field voltage of 2 V_pp_ with (red line) and without (blue line) a laser irradiation (Positive velocity means counter-field; negative velocity means co-field). Insets: (top left) Images of circular active motion in the counter-field direction. The images are accumulated for 10 frames and the time interval is 0.1 s. (bottom left) Images of circular active motion in the co-field direction. The images are accumulated for 6 frames and the time interval is 0.2 s. (top right) Image of EROT of a Janus particle which is facing with gold side up (up-down orientation).

The experimental setup is shown in Fig. 1 (see Materials and Methods). First, we measure the EROT of Au-silica Janus particles from 1 kHz to 4 MHz without the laser irradiation. We find that although the EROT of the Janus particles is mainly opposite to the rotating electric field (counter-field), the direction can be reversed to the same as the field (co-field) in the low-frequency region (<3 kHz), which is in agreement with the previous study^10^. To verify the effect of the thermal-electric coupling field, we measure the EROT of particles with laser irradiation. The thermal gradient is generated by higher absorption of the laser irradiation at the gold-hemisphere of the Janus particle. The EROT spectra with and without the laser irradiation are shown in Fig. 2. The error bars in the figure correspond to individual measurements of 10 distinct particles. In contrast to the normal EROT of Janus particles, a directionally circular motion of Janus particles with their silica hemisphere in forwarding direction is observed in a specific frequency region ranging from 1 to 6 kHz (the left region of the dotted line of Fig. 2). This circular motion of Janus particles can only be observed by simultaneously applying a thermal gradient and a rotating electric field. Without laser irradiation, only pure electrorotation of the Janus particles can be observed^10^. The circular motion can be divided into two parts: (i) translational motion from the electrically coupled self-thermophoresis of Janus particles, which dominates the speed of the circular motion and (ii) rotation from EROT of Janus particles, which dominates the direction of the circular motion. The circular motion follows the EROT direction of the Janus particles, which is co-field below 2 kHz but counter-field from 4 to 6 kHz (see Supplementary Movies S1 and S2). Note that in normal circular motion, both central force and tangential force are essential. However, the moving direction of self-thermophoresis is determined by its orientation. And the orientation of the Janus particle is determined by the electrorotation effect. Therefore, the central force is not necessary.

When the frequency exceeds 7 kHz, the motion of Janus particles transits from circular motion to normal EROT. In addition, the angular velocity of EROT under laser irradiation is smaller than that in the normal EROT. This could be attributed to the friction between the particles and the upper surface of the chamber due to laser irradiation^3^. We also observe that the gold-coated side of the Janus particle tends to face up (up-down orientation) and the orientation of particles could be changed by laser at larger frequencies, as shown in the top right inset of Fig. 2. The up-down orientation of particles is dominated by the electric field induced aligned force and light pressure from the laser illumination. The electric field induced aligned force leads the induced dipole on the particle to become parallel to the direction of the electric field and this becomes strong in the high frequency region due to a weakening in the screening ability (ability to screen the effect of the electrical induced dipole)^22^. The light pressure of the laser also pushes the Janus particle to the upper surface of the chamber and turns the gold-coated side to face up^3^. Thus, the orientation of the particles tends to up-down in the high frequency region when the laser illumination and the rotating electric field are applied simultaneously.

### Electrically enhanced self-thermophoresis of Janus particles

**Figure 3:**
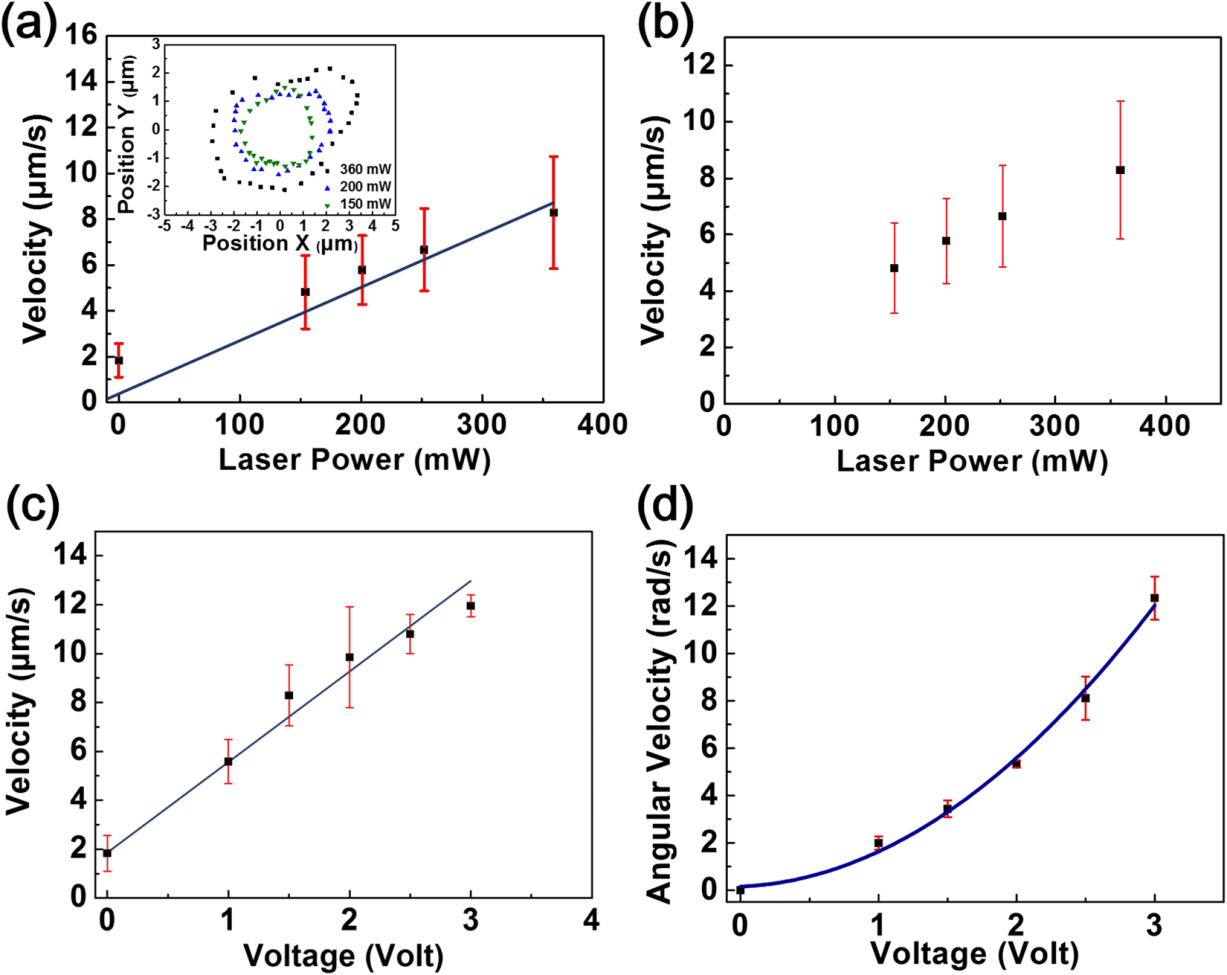
(**a**) The relation of the linear velocity of circular motion and laser power at a field frequency of 2 kHz and field voltage of 1.5 Vpp. Inset: The trajectories of the circular motion in the co-field direction, the radiuses of which are linearly proportional to laser power. The time interval of each point is 0.07 s. (**b**) The relation of the angular velocity of circular motion and laser power, which is independent of laser power. (**c**) The relation of the linear velocity of circular motion and voltage at a field frequency of 2 kHz and with a laser irradiation of 360 mW. (**d**) The relation of the angular velocity of circular motion and voltage, which is proportional to the square of voltage.

To obtain the thermal gradient dependence on circular motion, the circular motion of the Janus particles is measured with different laser powers at a constant frequency of 2 kHz and at a constant voltage of 1.5 V_pp_. The temperature profile is directly measured using a temperature sensitive dye, 2′,7′-Bis-(2-carboxyethyl)-5-(and 6)-carboxyfluorescein (BCECF) for a 2.34 μm Au-silica Janus particle fixed to the substrate and subjected to laser heating^23^. The temperature profile is shown in Supplementary Fig. S1. The temperature on the coated side is about 0.15 to 0.4 K higher than that on the other side (estimated by the temperature gradient around the particle) corresponding to laser power in the range of 150–360 mW. In the circular motion region (1 kHz to 6 kHz), the linear velocity of the circular motion is proportional to laser power but the angular velocity is insensitive to it, as shown in Fig. 3(a and b). We also measure the circular motion of Janus particles at different voltages. The result shows that linear velocity is roughly proportional to the strength of the electric field with an offset, and the angular velocity increases with the square of the field strength, Fig. 3(c and d). The increase of angular velocity can be explained by electrorotation. The increase of linear velocity with the strength of the electric field under a self-generated thermal gradient is regarded as electrically coupled self-thermophoresis.

**Figure 4:**
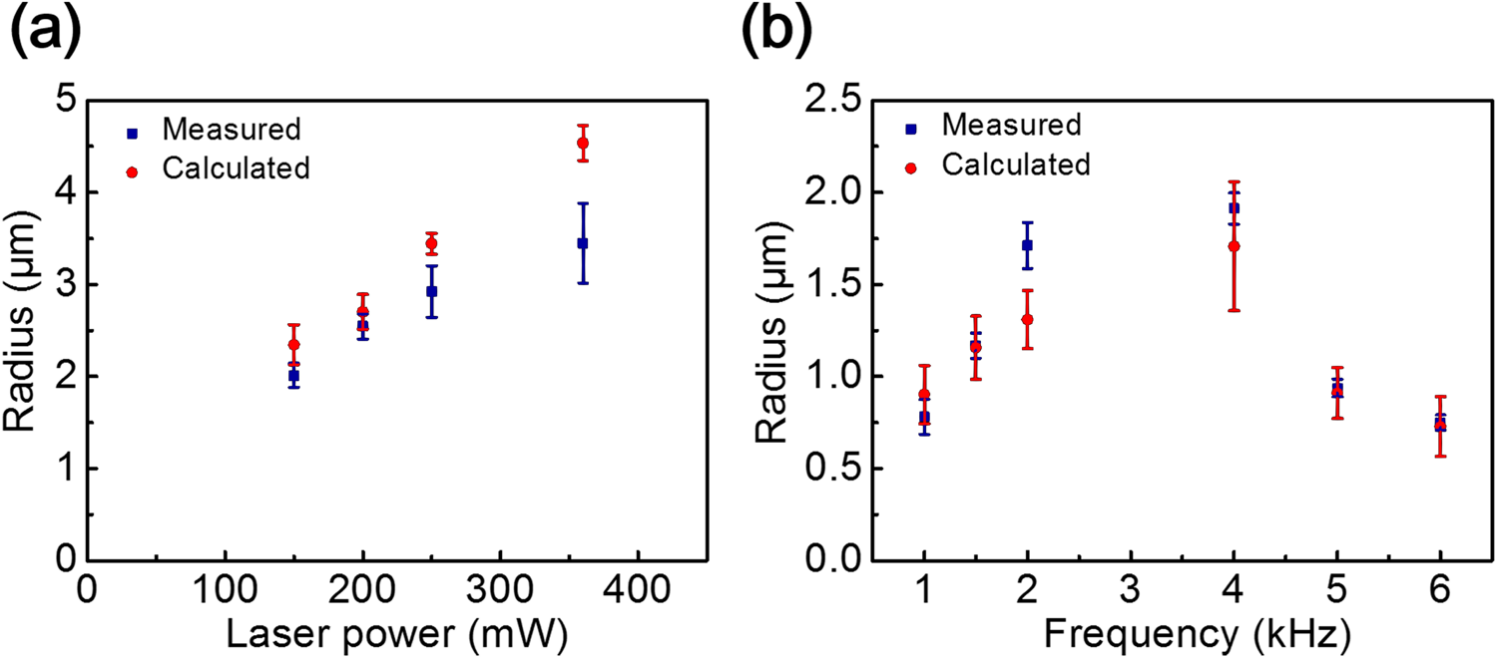
The comparison between calculated and measured radiuses under different (**a**) laser power; (**b**) frequencies.

**Figure 5:**
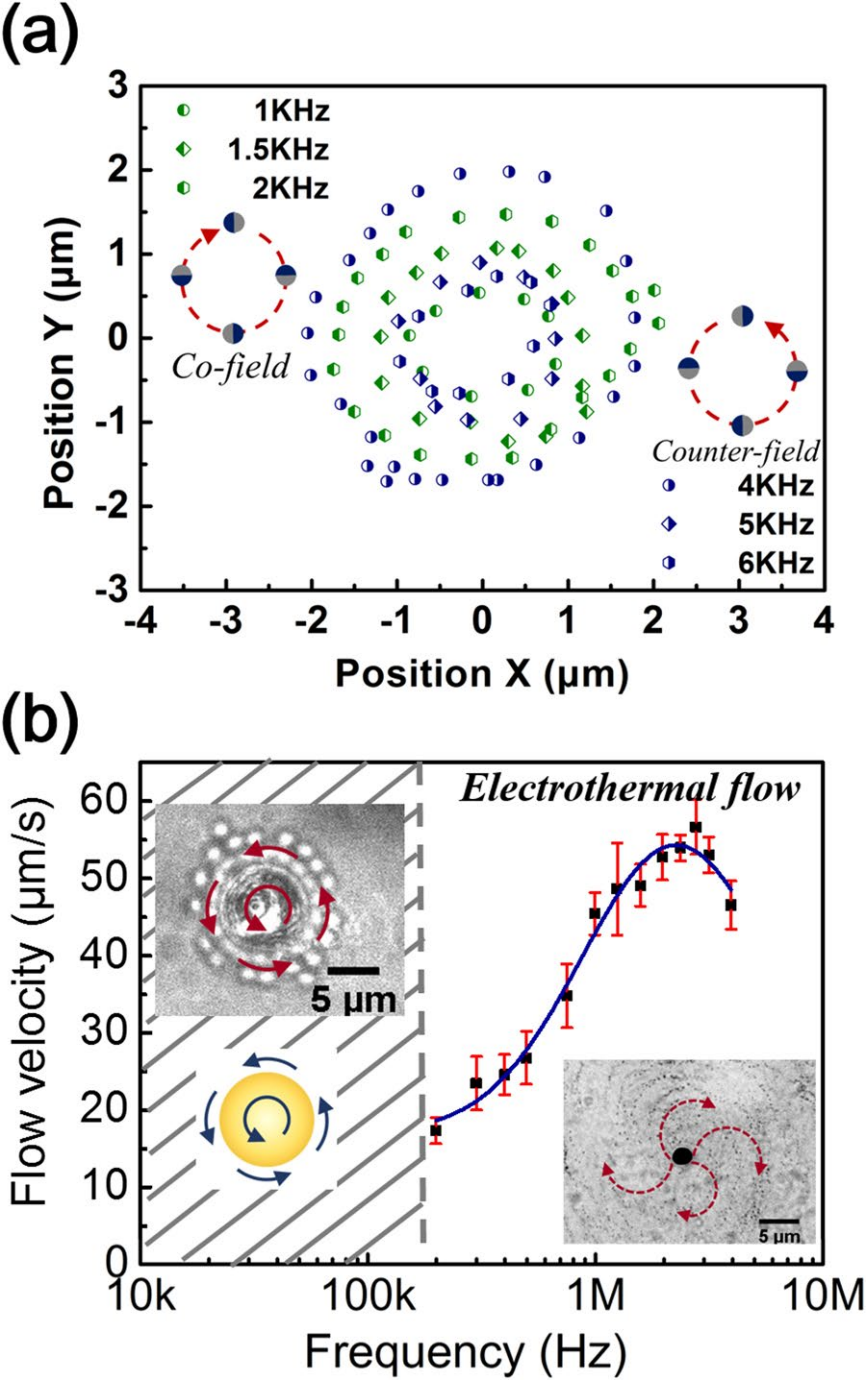
(**a**) The trajectories of the circular motion in the co-field (green) and counter-field (blue) direction at a field voltage of 2 V_pp_ and with a laser irradiation of 360 mW. The radiuses of the trajectories vary with field frequency depending on the angular velocity of the circular motion. The time interval of each point is 0.07 s. (**b**) The velocity of electrothermal flow measured at a radius about 5 μm from the particle center gradually increases with field frequency from about 200 kHz to 2 MHz. Insets: (right) The flow pattern of rotating electrothermal flow. The images are accumulated for 50 frames and the time interval is 0.0068 s. (left) Image and schematics of the flow around the particle resulting from the momentum transference of electrorotation from about 10 kHz to 200 kHz.

We also find that the radius of the circular motion trajectory increases with laser power, which also indicates that linear velocity is proportional to laser power, as shown in the inset in Fig. 3(a). The radius of the circular motion can be determined by the linear and angular velocity from $R=v/\omega$. We compare the calculated radius and the measured radius obtained from the particle trajectories. The results are shown in Fig. 4. This indicates that circular motion can be well separated into linear and angular motions. In Fig. 2, it can be seen that the angular velocity decreases with the frequency from 1 kHz to 2 kHz (co-field), but increases from 4 to 6 kHz (counter-field). Based on our observation, the radius of the circular motion trajectory with respect to the nearly invariant linear velocity is inversely proportional to the angular speed as shown in Fig. 5(a).

### The model of the electrically enhanced self-thermophoresis

According to the results, the circular motion could be divided into two parts: translational motion and rotational motion. The translational motion of Janus particles is dominated by their self-thermophoresis^3^. Based on the previous study^3^, the direction of the force follows the polarity of the Janus particle with the dielectric side forward. This force arises from the thermal gradient generated by the Janus particle itself due to laser absorption asymmetry. Thus, the force is always along the polarity of the Janus particle and there is no torque acting on the particle from this force. The translational velocity of the circular motion increases with Stokes drag force along the tangential direction, which should be in accordance of the thermophoresis model. The rotational motion of Janus particles is dominated by the their EROT, as reported in the previous study^10^. There are several models proposed to explain the thermophoresis of colloidal particles under different conditions, such as depletion force^24^, thermoelectric effect^17^. Specifically, “*Electric double layer model*” is proposed to explain the thermophoresis of the charged particles in the aqueous solution^15,25,26^. To elucidate the mechanism of the active circular motion, we consider the “*Electric double layer model*” of thermophoresis, which indicates that the density of positive and negative charges within the electric double layer is re-distributed by the thermal gradient. This re-distribution of charges results in excess hydrostatic pressure that drives the particles under the thermal gradient. The thermophoretic velocity is given as^15,27^,

1 $$u=-\,\frac{\varepsilon {\zeta }^{2}}{3\eta }\frac{{\nabla }T}{T}$$

where *ε* is the permittivity of fluid, ζ is zeta potential, *η* is viscosity, *T* is temperature, and *∇T* is the thermal gradient. However, it is difficult to directly compare the theoretically predicted values of thermophoretic velocity from equation (1) with the experimental values. There are additional factors, such as boundary conditions at the interface between the solid and liquid^24^ and interactions between the colloid and liquid^28,29^ that result in a difference between the theoretical and experimental values. However, using equation (1), the relative variants of the thermophoretic velocity resulting from the various thermal gradients and induced zeta potential can be estimated. Equation (1) shows that the thermophoretic velocity depends on the square of the zeta potential. In an external electric field, the zeta potential can be written as^19,20^,

2 $${\zeta }_{t}={\zeta }_{0}+{\zeta }_{i}(E)$$

where *E* is the strength of the electric field, ${\zeta }_{t}$ is total zeta potential, ${\zeta }_{0}$ is intrinsic zeta potential in the equilibrium system and ${\zeta }_{i}(E)$ is the induced zeta potential^19^. Equation (2) shows that zeta potential can be enhanced by an external electric field. According to a previous study by Bazant *et al*.^30^, induced zeta potential on the metal hemisphere of Janus particles is given as,

3 $${\zeta }_{i}(E)={\zeta }_{c}+\frac{3}{2}{Ea}({\alpha }\,\cos \,\theta +\beta \,\cos \,\varnothing \,\sin \,\theta )$$

where *αE* is strength of the electric field in the $\theta =0\,(or\,\hat{x})$ direction, $\beta E$ is the strength of the electric field in the $\theta =\pi /2,\varnothing =0\,(or\,\hat{y})$ direction, *a* is particle radius and ${\zeta }_{c}=-\,(3/4)\alpha Ea$ satisfies the total charge constraint. From equations (1) and (2), thermophoretic velocity is proportional to the thermal gradient and the square of the electric field strength with an offset due to the intrinsic zeta potential, ${\zeta }_{0}$. The effect of thermal-electric coupling field for thermophoresis of colloidal particles could be estimated by this model.

### Frequency responses under thermal-electric coupling field

In the proposed model, the formation of an electric double layer is essential for the electrically enhanced self-thermophoresis. The charging time scales of our system are considered as ${\tau }_{e}^{-1}\le \omega \le {\tau }_{p}^{-1}$ ^5,19^. The upper limit is the characteristic “*RC* time”, ${\tau }_{p}=\lambda a/D$, for the formation of the electric double layer on the particles, where *D* is the ion diffusion coefficient and $\lambda$ is the Debye length. The lower limit is the charging time of the electrodes, ${\tau }_{e}=\lambda L/D$, where 2 *L* is the distance between electrodes^5,19^. In our system, the lower and upper limits are ${\tau }_{e}^{-1}\approx 600\,Hz$ and ${\tau }_{p}^{-1}\approx 85\,kHz\,$^19^. The frequency region of active circular motion (1 kHz to 6 kHz) is indeed in this band of charging frequency. However, when the frequency increases above 7 kHz, the circular motion disappears and the only electrorotation can be observed. This could be attributed to the up-down orientation of Janus particles and the gradual increase in angular velocity.

We also measure the response of Janus particles and their surrounding fluid at other frequencies. In the pure EROT domain (from 10 to 200 kHz), we observe that the flow around the particles resulting from the momentum transfer from the Janus particle EROT, as shown in the left inset of Fig. 5(b). On the other contrary, in the higher frequency region (above 700 kHz), electrothermal flows around the particles gradually dominate with the field frequency^13^, Fig. 5(b).

## Discussion

There are several possible mechanisms for the circular motion of Janus particles, such as dielectrophoresis (DEP), ICEP, optical tapping, and electrothermal flow induced particles motion. Firstly, DEP is a kind of electrokinetic phenomenon resulting from the induced dipole on the polarized particle interacting with a non-uniform electric field. The DEP response of particles should be attracted to (positive-DEP) or repelled by (negative-DEP) the edge of the electrodes rather than the circular motion as we observed. Moreover, the linear velocities of the circular motion are measured from the particles locating at the central region of the four-phase electrode array and the DEP response should be small enough to neglect. Secondly, ICEP of Janus particles mainly results from asymmetric ICEO slip flow along the tangential direction of the particle surface^5^. However, the circular motion of Janus particles in our system could only be observed by applying a thermal gradient and a rotating electric field at the same time. By applying only a rotating electric field, only pure electrorotation of Janus particles is observed (without ICEP motion)^10^. Furthermore, ICEO is uniformly inward with respect to polarizable objects under a rotating electric field^31^. Thus, the uniformly inward ICEO along the radial direction of the particle surface shouldn’t result in the ICEP of particles. The other possible mechanism is electrothermal flow induced motion of particles. However, electrothermal flow can only be generated at the high frequency region^13^ and thus this possibility should be excluded. The laser would apply additional confinement due to scattering force from light. This force would only make the Janus particle stay in the center region^3^ depending on the diameter of the laser irradiation region.

We also compare the difference between our results with the proposed model. In the experiments, a linear relation between the velocity and voltage is observed, as shown in Fig. 3(c). Compared with the ideal condition of a free moving particle used in the theory, the particle is moving on the upper surface of the chamber and tends to face up when the voltage increases as shown in Fig. 2 inset. Thus, the difference between equation (1) and the velocity-voltage relation observed in experiments may result from the change of the particle orientation and the particle-wall friction^5^. By fitting the mean square displacement, ${MSD}=4{Dt}+{V}^{2}{t}^{2}\,$^3,4^, we obtain the ratio of the diffusion coefficient of particles at different voltages, $({D}_{1V}/{D}_{2V})\approx 2$ (Supplementary Fig. S2), which may support the increase in the particle-wall friction. The relative magnitudes from the electric field and thermal gradient with the proposed model are also estimated. The thermal gradient induced by the particle under laser irradiation is 0.075 to 0.2 K/µm corresponding to the applied laser power of 150 to 360 mW and the induced zeta potential is 15.6 to 46.8 mV corresponding to the applied voltage of 1 to 3 V. According to equation (1), the linear velocity of the particle increases about 2.7 times due to the thermal gradient and increases about 2.2 times due to the electric field with estimating the value of the intrinsic zeta potential as approximately 40 to 50 mV. The effects of the thermal gradient and induced zeta potential are of a similar magnitude as we observed.

To conclude, we report the circular self-propulsion of Janus particles by applying laser irradiation and a rotating electric field concurrently in low field frequency region (1 to 6 kHz). The direction of active motion could be co- or counter-field depending on the frequency of the electric fields. We propose that self-thermophoresis could be electrically enhanced by induced zeta potential resulting in a tunable active motion of Janus particles. The manipulation of active motion under a thermal-electric coupling field may provide a new way to power and control micro-machines.

## Materials and Methods

To prepare the Janus particles, a monolayer of 2.34 µm silica particles is prepared by a drying process, put into a sputter and coated with 35 nm thick gold^10,32^. An image of the 2.34 µm Au-silica Janus particles is shown in Fig. 1(a). A thin chamber, containing a solution and particles, is sandwiched between a cover glass (bottom) and a four-phase indium tin oxide (ITO) coated glass electrode (top) with a 20 μm spacer. To prepare a four-phase electrode, the ITO conductive layer is removed by a fiber laser marking machine selectively (1,064 nm, 20 W, 90- to 120-ns pulse width, and 20- to 80-kHz pulse repetition frequency)^32^. The distance between the diagonal electrodes is 150 μm. To generate a rotating electric field, the electrodes are connected to a 2-channel function generator and two invertors, and the phase shift between the adjacent electrodes is set at 90 degrees^10,31,32^, as shown Fig. 1(b). A laser (Nd:YAG, 1064 nm) is fed from the bottom of the chamber through an oil immersion objective (100×, NA 1.3). The laser is defocused to irradiate a wider area about 15~20 μm in diameter, as shown Fig. 1(c). The power of the laser is varied from 0 to 360 mW before the objective. Images of the particles are collected through the same objective by a CMOS camera at 120 frames per second.

## Electronic supplementary material

Supplementary information

Movie S1

Movie S2
