# Supplementary material for: Electrically Enhanced Self-Thermophoresis of Laser-Heated Janus Particles under a Rotating Electric Field
Source: Sci Rep. 2018 Apr 13;8:5945. doi: 10.1038/s41598-018-24343-w (PMC5899123; doi:10.1038/s41598-018-24343-w)

Electrically Enhanced Self-Thermophoresis of Laser-Heated Janus Particles under a Rotating Electric Field: Supplementary information

*Yu-Liang Chen, Cheng-Xiang Yang and Hong-Ren Jiang**

Movie S1: Real-time circular motion of a 2.34 μm Au-silica Janus particle in the counter-field direction. The movie is taken at the condition that the laser power is 360 mW, the voltage of an AC electric field is 1 V, and the frequency of the electric field is 5 kHz, measure before the objective.

Movie S2: Real-time circular motion of a 2.34 μm Au-silica Janus particle in the co-field direction. The movie is taken at the condition that the laser power is 360 mW, the voltage of an AC electric field is 1.5 V, and the frequency of the electric field is 2 kHz, measure before the objective.

Figure S1: The temperature profile measured by a 2.34 μm Au-silica Janus particle fixed on the substrate and subjected to laser heating with 160 mW. Inset: The measurement of the temperature profile (orange cone is the defocused laser beam).

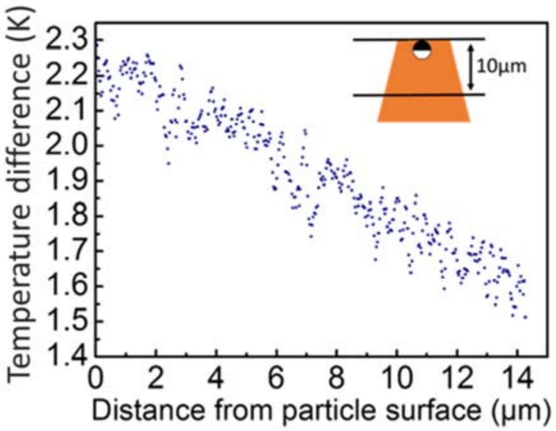

Figure S2: The mean square displacement of the particle under 1V and 2V at a field frequency of 2 kHz and with a laser irradiation of 360 mW. By fitting the mean square displacement with V_{1v}=5.6 μm/s and V_{2v}=9.9 μm/s obtained from Fig. 3(c), diffusion coefficient of particles at 1V and 2 V are 0.74 μm^2/s and 0.41 μm^2/s respectively.

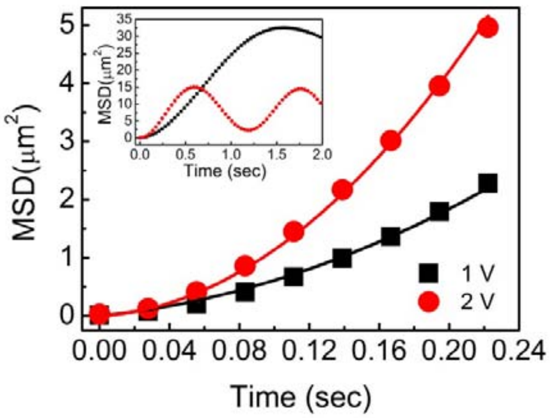

Supplement: Supplementary file 1 — Supplementary information [file 41598_2018_24343_MOESM1_ESM.pdf]
